# Water Ultrasound-Assisted Extraction of Polyphenol Compounds from Brewer’s Spent Grain: Kinetic Study, Extract Characterization, and Concentration

**DOI:** 10.3390/antiox9030265

**Published:** 2020-03-23

**Authors:** Patricia Alonso-Riaño, María Teresa Sanz Diez, Beatriz Blanco, Sagrario Beltrán, Ester Trigueros, Oscar Benito-Román

**Affiliations:** Department of Biotechnology and Food Science (Chemical Engineering Section), University of Burgos, 09001 Burgos, Spain; pariano@ubu.es (P.A.-R.); bblanco@ubu.es (B.B.); beltran@ubu.es (S.B.); etrigueros@ubu.es (E.T.); obenito@ubu.es (O.B.-R.)

**Keywords:** brewer’s spent grains, ultrasound assisted extraction, polyphenol compound, extractives concentration

## Abstract

Brewer’s spent grain (BSG) was chemically characterized obtaining 52.1% of carbohydrates, 17.8% protein, 5.9% lipids, 13.5% insoluble lignin and 24.3% of water-soluble extractives. This work has been focused on the study of polyphenol extraction of the extractive fraction by water ultrasound-assisted extraction. Selected extraction conditions were 47 °C and 21.7 mL water/*g_dry-BSG_*. The effect of solvent polarity on polyphenol extraction was studied by using ethanol aqueous mixtures, from 20% to 100% ethanol. The kinetics of polyphenol extraction have been fitted to the power law and the Weibull models yielding mean values of the root mean square deviation lower than 7.5%. Extracts have been characterized in terms of quantification of individual phenolic compounds by HPLC-DAD and protein and sugar soluble fractions (glucose, xylose, and arabinose). Polyphenol profile has been compared with other hydrolytic techniques, such as acid, basic and enzymatic hydrolysis, showing that ultrasound was not as effective as basic hydrolysis to release the phenolic acids esterified to the cell wall. A further centrifuge ultrafiltration concentration step was able to yield a retentate enriched in the protein fraction while individual phenolic compounds where mainly transferred to the permeate.

## 1. Introduction

Brewer’s spent grain is the main by-product produced by breweries, accounting for 20 kg per 100 L of beer produced [1]. BSG represents up to 30% of the starting malted grain [2]. Currently, it is mostly used as animal feed, but its high moisture content results in an increased cost of transportation. Furthermore, the presence of fermentable sugars, reduces its shelf life. However, BSG is a lignocellulosic material rich in proteins, polysaccharides and other bioactive compounds such as polyphenols. Therefore, BSG presents a great potential to obtain valuable products to be incorporated in the food and pharmaceutical industries. Specially, taking into account that European beer production reached nearly 39.7 billion liters in 2017 [3]. Furthermore, BSG valorization will contribute to reduce the percentage of BSG that is released in landfills yearly.

This work is mainly focused on the BSG extractives valorization by solvent extraction optimization and further centrifuge ultrafiltration concentration. According to the literature, the amount of extractives from different BSG sources were found to vary between 10.7 and 14.4 g/100 g in a dry basis [4,5]. Valorization of phenolic compounds found in the extractive fraction, offers a great potential due to their antioxidant properties. Most of the phenolic compounds of the barley grain are contained in the coat-pericarp-husk layer [2]. Therefore, BSG can be regarded as a potentially valuable source of these compounds, mainly hydroxycinnamic acids [6]. 

Solvent extraction is the primary and essential step to recover the bioactive compounds from the raw material. Extraction efficiency is determined by different factors such as type of solvent, temperature, pH, solvent to solid ratio, particle size and extraction method. In this work, the potential of Ultrasound Assisted Extraction (UAE) was explored to obtain a rich fraction of polyphenol extractives and compared with other hydrolytic techniques. Ultrasound has been proposed as a key technology to reach higher extraction yields and faster extraction kinetics than conventional extraction processes. Chemat et al. [7] reviewed the mechanisms involved for an improved extraction process of natural products, namely fragmentation of the raw material, erosion, sonocapillary effects, sonoporation, shear stress, and detexturation among others.

The influence of the extraction solvent on the recovery of phenolic compounds from BSG has been reported in the literature [4]. Acetone aqueous mixtures (60% *v*/*v* acetone) at 60 °C (1 g BSG/20 mL solvent for 30 min) was the best solvent for phenolic compounds extraction with 9.9 ± 0.41 mg gallic acid equivalent/*g_dry BSG_*. By using water as extraction solvent (T = 80 °C), these authors reported no statistically significant difference in the total phenolic compounds compared to methanol and ethanol aqueous mixtures (less than 20% and 60% *v*/*v* water content for ethanol and methanol aqueous mixtures respectively and extraction temperature of 60 °C). 

Based on these results, one of the main objectives of this work was to provide a comprehensive study of total polyphenol content (TPC) extraction kinetics from BSG by using water as solvent, having the advantage of being “the greenest solvent”. Enhanced extraction kinetics have been explored by using UAE and results have been compared with mechanical stirring extraction. The effect of particle size, solvent to solid ratio and solvent polarity on the TPC extraction kinetics has been analyzed. TPC extraction kinetics were fitted to different empirical models as proposed by Kitanovic et al. [8]. Individual phenolic compounds were identified, quantified and compared with other hydrolytic methods, such as enzymatic, basic, and acid hydrolysis. Other components present in the extracts such as soluble proteins were also measured. Finally, a further concentration step was performed by centrifuge ultrafiltration.

## 2. Materials and Methods 

### 2.1. Raw Material

BSG samples were obtained from Brebajes del Norte S.L. (Dolina, craft beer) a local brewery factory in Burgos (Spain) with an initial moisture content higher than 80% *w*/*w*. Samples were water washed and dried in an air convection oven at 45 °C until a final moisture content of 8% *w*/*w*. Dry BSG was stored at 4 °C until use and it was used in the extraction study. To study the effect of particle size on TPC extraction kinetics, dry samples were ground in a *QILIVE*, 5321 miller. Particle size distribution of the original and ground feedstock was determined by a vibratory sieve shaker (*CISA*, RP.09) and is presented in Table 1. A total of 65.9% *w*/*w* of ground BSG presented a particle size lower than 0.5 mm, while for the original feedstock, about 85% *w*/*w* of the particles presented a size between 2 and 4 mm. 

### 2.2. Biomass Characterization

BSG chemical composition was determined according to the NREL standard protocols to determine structural carbohydrates (cellulose and hemicellulose), lignin, moisture, total solids, ash, total extractives, and protein [9].

Starch analysis was carried out using the “Total starch assay kit” (Megazyme, Wicklow, Ireland).

### 2.3. Mechanical Stirring and Ultrasound Assisted Extraction

For both extraction methods, a specific amount of dry BSG was suspended in the selected solvent and extraction was performed at the desired extraction temperature for 24 h and 1 h in a mechanical stirring system and in the UAE extraction equipment, respectively. 

For UAE, a 750 W Sonics Material^TM^ with a 13 mm probe was used. Samples were processed at a constant ultrasound frequency of 20 kHz. The extraction temperature was controlled by circulating water through the external jacket of the extraction vessel connected to a thermostat, which was fixed to different temperatures depending on the final working temperature. The BSG sample and the solvent were introduced in the thermostated vessel (Φ = 4.8 cm, V = 199 cm^3^) and the probe was submerged in the mixture at a constant depth of 2 cm from the bottom of the vessel. The amplitude was fixed at 100% and the temperature and energy input were recorded along the extraction experiments. UAE was performed in pulse mode (5 s on and 5 s off). Total treatment time under pulsed conditions, t_p_, was determined as:

t_p_ = t_c_ (1+(1/R)) (1)

where t_c_ is the corresponding time of exposure in a continuous experiment, 30 min, and R is the ratio on/off. The ultrasonic Power Density, PD (J/s·g), was evaluated as:

(2) $$PD=\frac{E}{t\cdot m}=\frac{P}{m}$$

where *E*, is the energy input (J), *t* is the ultrasonication time (s), *P*, the ultrasonic power (J/s = W) and *m* is the BSG mass (g). Based on equation 2, by varying BSG mass, at fixed amplitude, the power density was also varied and its effect on polyphenol extraction was analyzed.

The effect of particle size on TPC extraction kinetics have been determined by mechanical stirring extraction and UAE by using deionized water as solvent. Other operating variables were also studied, such as the solvent volume to BSG mass ratio (*v*/*w*) in the range from 35.3 to 10.9 mL/g_BSG, dry_, temperature (39–58 °C) and solvent polarity by performing the UAE with different ethanolic aqueous mixtures as solvent. 

Results were expressed as extraction yield, defined as mg of gallic acid equivalent (GAE)/*g_BSG,dry_*. Results were also compared in terms of productivity (*Pr*), being the yield obtained in a certain range of time, △t:

(3) $$Productivity,\;Pr=\frac{mg\;GAE}{g_{BSG,dry\;}\Delta t}$$

### 2.4. Hydrolysis Treatments

The release of TPC by UAE has been compared with enzymatic, acid and basic hydrolysis. Acid hydrolysis was performed similar to Arranz and Saura Calixto [10]. Briefly, 200 mg of dry BSG were incubated with a mixture of 20 mL of methanol and 2 mL of concentrated sulphuric acid for 20 h at 85 °C. Basic hydrolysis was carried out as previously described by Benito-Román et al. [11]. Two grams of dry BSG was digested with 40 mL of NaOH 2 M for 4 h at 65 °C. The mixture was acidified to pH 2–3 by the addition of hydrochloric acid and centrifuged. Enzymatic hydrolysis was carried out by incubating the BSG with different amounts of the commercial preparation of xylanase from *Trichoderma Longibrachiatum* (Biocon Española, S.A.) at different concentrations 1%, 3%, and 6% *w*/*w* referred to the BSG mass for 24 h. Temperature and solvent to BSG mass ratio (*v*/*w*) were fixed at 47 °C and 21.7 mL water: *g_BSG,dry_*, respectively. Samples were collected and stored at –18 °C until analysis. 

### 2.5. Total Polyphenol Content (TPC) and Antioxidant Capacity

TPC were determined by using the Folin–Ciocalteu reagent (VWR). One hundred microliters of the BSG extract were mixed with 2.8 mL of water, and subsequently with 100 µL of the Folin–Ciocalteu reagent. After that, 2 mL of sodium carbonate 7.5% (*w*/*v*) were added and the reaction started. Absorbance was measured after 60 min of reaction at 750 nm. A blank was also prepared using water instead of the extract. A calibration curve was prepared with standard solutions of gallic acid by following the same colorimetric method and results were expressed as mg of GAE per gram of dry BSG.

The FRAP method was performed according to Benzie and Strain [12]. 2850 μL of the working FRAP reagent were added to 150 μL of the extract and incubated at 37 °C for 30 min. Absorbance was read at 593 nm. As standard, a solution of FeSO_4_·7H_2_O (0.1 M) was used. Results were expressed in µmoles of Fe^2+^ per gram of dry BSG.

### 2.6. Identification and Quantification of Extracts Components

Chromatographic separation was performed on HPLC/DAD Agilent 110 with a Kinetex^®^ 5 µm Biphenyl 100 Å, 250 × 4.6 mm column (Phenomenex). The mobile phase consisted of (A) ammonium acetate 5 mM with acetic acid (1%; *v*/*v*) in water and (B) ammonium acetate 5 mM with acetic acid (1%; *v*/*v*) in acetonitrile. The gradient profile was the following: from 0 to 7 min 2% of solvent B (isocratic), from 7 to 20 min from 2% to 8% solvent B, from 20 to 35 min from 8% to 10% solvent B and from 35 to 55 min 10% to 18% solvent B and post time of 10 min. The flow rate was set to 0.8 mL/min and temperature column was 25 °C. UV detection was done at 240, 280, 330, 340, 350, and 370 nm. Before injection, extracts were filtered through 0.45 μm pore size. HP ChemStation software was employed to collect and analyze the chromatographic data delivered by the diode array detector and own library was used to identify the different polyphenols by comparing retention times and spectral data with those of authentic standards. p-Hydroxybenzoic acid, protocatechuic aldehyde, syringic aldehyde, vanillic acid, vanillin, syringic acid, p-coumaric acid, ferulic acid, and sinapic acid standards were purchased from Sigma-Aldrich. Standard solutions were prepared by dissolution of the compound in methanol.

Identification and quantification of glucose, xylose and arabinose were performed by HPLC-RID Agilent 1260 with an Aminex HPX-87H column (300 × 7.8 mm, Bio-Rad) using H_2_SO_4_ 10 mM as mobile phase with a flow rate of 0.6 mL/min. The column and detector were maintained at 40 °C. 

Protein content was determined by total nitrogen measured by a Shimadzu TOC-V-CSN analyzer with a conversion factor of 6.25.

### 2.7. Solvent Extraction Kinetics

TPC extraction kinetic curves for the different extraction methods and extraction conditions were fitted to different empirical models described and collected by Kitanovic et al. [8]. Among them, in this work, the power law model and the Weibull model were considered.

#### 2.7.1. Power Law Model

The power law model has been proposed to describe the extraction mechanism of any compound through non-swelling material:

(4) $$Extraction\;yield,\;mg\;GAE/g_{BSG,dry}=Bt^{n}$$

where *t* is extraction time, *B* is a constant incorporating the characteristics of the particle-active substance system and *n* is the diffusional exponent, with values lower than 1 for most vegetable materials. This model does not approach to a limit with time.

#### 2.7.2. Weibull’s Model 

The Weibull model was expressed as:

(5) $$Extraction\;yield,\;mg\;GAE/g_{BSG,dry}=A\left(1-\exp\left(-kt^{n}\right)\right)$$

where *t* is the extraction time, A is a kinetic parameter that represents the maximum extraction yield at infinite extraction time and k is a kind of extraction rate constant. The exponent *n* is the shape parameter of the extraction curve. If *n* > 1, the curve is sigmoidal with upward curvature, and if *n* < 1, the curve is parabolic with a high initial slope followed by an exponential shape.

To estimate the kinetic parameters, non-linear regression was performed by using the Marquardt algorithm (Statgraphics X64). Experimental results were then compared with those of the model prediction through the values of the Root Mean Square Deviation (RMSD) between experimental and calculated extraction yield:

(6) $$RMSD=\sqrt{\frac{\sum_{i=1}^{n}{\left(Yield_{exp}-Yield_{calc}\right)}^{2}}{n}}\times 100$$

where *n* is the number of experimental data points in each extraction.

### 2.8. Centrifuge Ultrafiltration

Aqueous extracts obtained by UAE were treated by centrifuge ultrafiltration by using Amicon Ultra centrifugal filters with different Nominal Molecular Weight Limit (NMWL): 3000, 10,000, and 100,000 Dalton. The regenerate cellulose membrane of the filters allowed high recovery. A ratio of permeate:retentate volume of 3:1 mL was fixed. Protein, sugars, and polyphenols content was determined, and the retention ratio was evaluated as:

(7) $$R=1-\frac{C_{i,p}}{C_{i,f}}\times 100$$

where *C_i,p_*, and *C_i,f_* are the concentrations in the permeate (*p*) and initial extract (*f*), respectively, and *i* refers to polyphenol compounds, protein or sugars. The yield (*Y*) in the permeate and retentate (*r*) was evaluated as a mass (*m*) ratio [13]:

(8) $$Y\left(\%\right)=\frac{m_{i,p-r}}{m_{i,f}}\times 100$$

### 2.9. Statistical Analysis

Statistical difference and correlation coefficient between total polyphenol compounds and antioxidant capacity were obtained using the software Statgraphics X64. The results are presented as the mean ± standard deviation of at least three replicates. The significance of the differences was determined based on an analysis of the variance with the Fisher’s Least Significant Difference (LSD) method at *p*-value ≤ 0.05.

## 3. Results and Discussion

### 3.1. Biomass Characterization

Table 2 lists the BSG composition according to the NREL protocols. The polysaccharide fraction (starch, cellulose and hemicellulose) accounts for 52.1 ± 3.6% in a dry weight basis. The local BSG used in this work showed an important amount of starch, 7.87% *w*/*w* (78.7 mg starch/g dry BSG), that can be associated with the source of the BSG. Robertson et al. [14] reported that the starch content of BSG from lager and ale producers shows significant differences. These authors reported medium values around 97.6 mg starch/g BSG and 35.8 mg starch/g BSG for ale and larger producers respectively.

According to the two-step extractives determination proposed in the NREL, extractives of BSG were found to be 26.03 ± 0.98% (*w*/*w*) with a TPC of 2.72 ± 0.04 and 1.61 ± 0.15 mg GAE⁄*g_BSG,dry_* for water and ethanol extractives respectively. 

### 3.2. Kinetics of Extraction of Phenolic Compounds from BSG

Taking into account the water extractive fraction of the BSG, valorization of its polyphenol fraction was studied by ultrasound assisted and conventional extraction by using water as solvent.

#### 3.2.1. Comparison of UAE and Mechanical Stirring Extraction

**Figure 1:**
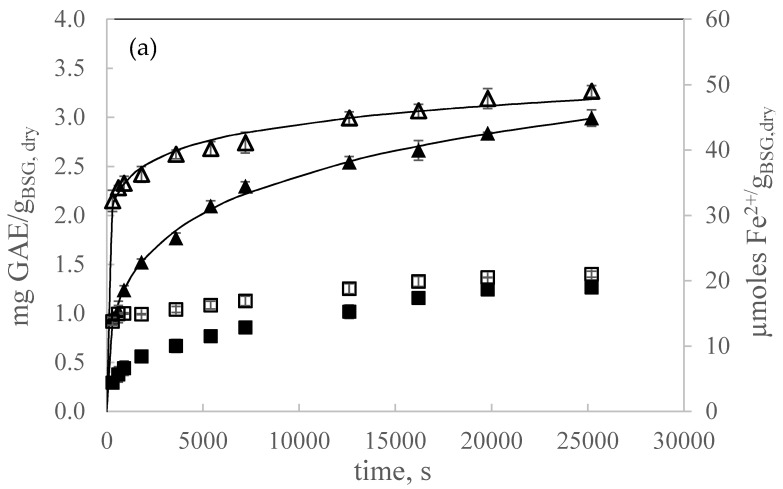

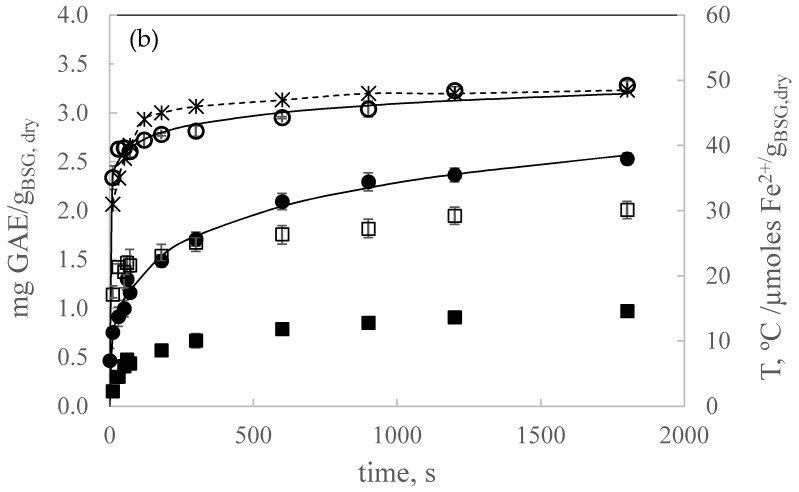
Total polyphenol content (TPC) water extraction kinetics at 47 °C and 21.7 mL:*g_BSG,dry_* (**a**) mechanical stirring extraction (Δ ground, ▲ non ground) (**b**) UAE (○ ground, ● non ground). The secondary axis represents the reducing power of the extracts as determined by the FRAP assay (□ ground, ■ non ground) and the temperature profile along UAE extraction (×). Continuous lines represent the Weibull model.

First, TPC extraction kinetics were determined by mechanical stirring extraction and UAE to assess the improvement of UAE (Figure 1a and Figure 1b, respectively). The temperature and solvent volume to dry BSG mass ratio (*v*/*w*) were fixed at 47 °C and 21.7 mL:*g_BSG,dry_*, respectively. Figure 1b also shows the temperature profile for a 30 min sonication time (60 min of total experiment) treatment in pulse mode at 100% amplitude (79 µm), for a temperature of the jacketed water of 30 °C. A sharp temperature increase was observed during the first three minutes of sonication, and then it reached a plateau with a mean value of 47 ± 1 °C. Similar temperature profiles have been reported in the literature for sonication treatments [15].

For both extraction methods, faster kinetics were obtained when using ground BSG due to enhanced internal mass transfer. Comparing UAE and mechanical stirring extraction, a significant improvement was observed by UAE observing much faster extraction kinetics, showing the effectiveness of UAE. For ground BSG, by mechanical stirring agitation, extraction was nearly completed after 24 h, but by UAE, extraction was completed after only 1200–1800 s. Comparing final extraction yields by the two extraction methods, an increase of 55% and 30% was observed for non-ground and ground BSG, respectively. It is clear that the improvement of UAE was less pronounced when using ground BSG particles. This fact was also observed by Galvan d’Alessandro [16] in the extraction of polyphenols from black chokeberry. These authors concluded that the positive effect of sonication was less evident, when the operating conditions were favorable for the extraction of polyphenols, as it has been also observed in this work.

Productivity at the final extraction time for both extraction methods was 0.109 mg GAE/*g_BSG,dry_*·min and 0.0078 mg GAE/*g_BSG,dry_*·min for UAE and conventional extraction, respectively. It is clear the higher productivity obtained by UAE. Furthermore, at 5 min of extraction time (300 s) 90% of the final extraction yield was already achieved by UAE, while, only 65% by mechanical stirrer agitation.

Figure 1a,b also show the antioxidant capacity along extraction time, as determined by the FRAP assay, observing in all cases an increase of the reducing power of the extract as the extraction yield of TPC increased. A multiple variable analysis was performed to determine the correlation between both variables yielding a positive correlation between them for *p*-values below 0.05 with a correlation coefficient of 0.9462.

#### 3.2.2. Effect of Solvent to Mass Ratio by UAE

**Figure 2:**
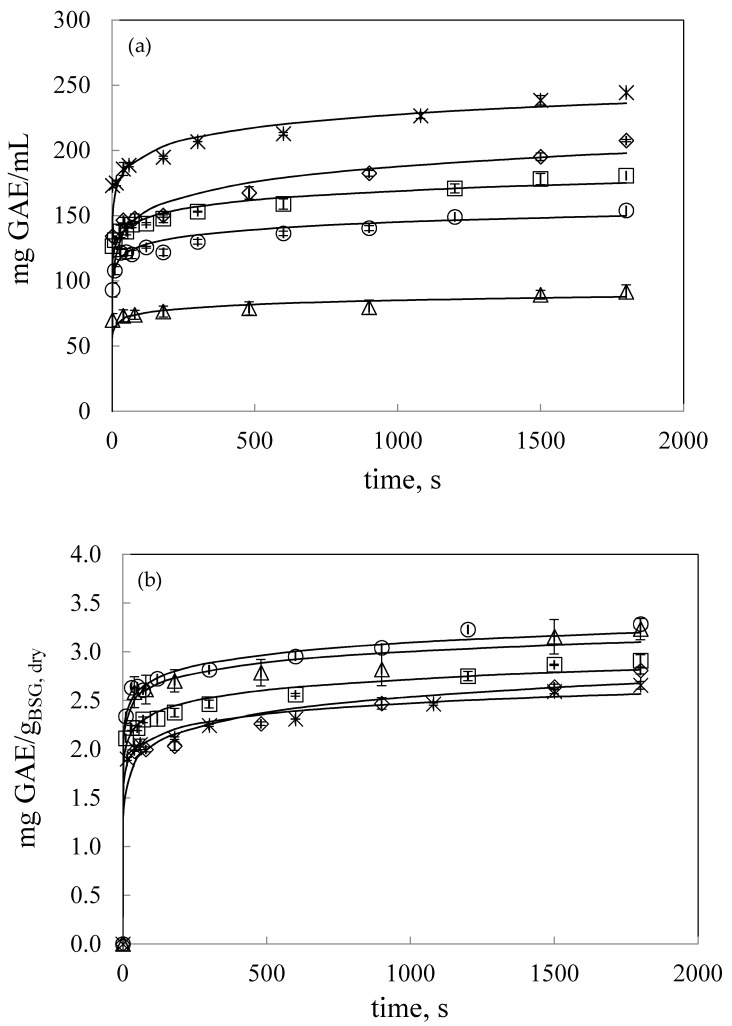
(**a**) Total polyphenol content in the water extracts as mg GAE/L as a function of time (**b**) Extraction yield, mg GAE/g_BSG,dry_ for UAE at 47 °C, at different solvent:mass ratios (mL:g_BSG,dry_) as a function of time: × 10.9; ◊ 13.6; □ 18.1; ○ 21.7; △ 35.3. Continuous lines represent the Weibull model.

**Figure 3:**
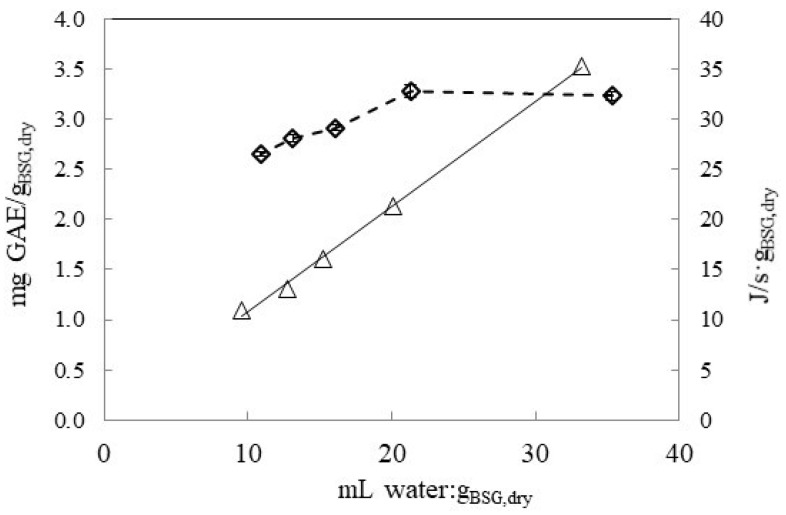
TPC water extraction yield, mg GAE/*g_BSG,dry_* (◇) and power density, △, (PD, J/s·g) as a function of solvent:mass ratio after 1800 s of UAE at 47 °C. Continuous line represents the linear fitting of PD (PD = 1.0547 (mL:g) + 0.1905, R^2^ = 0.9983).

The effect of solvent to dry mass BSG ratio (*v*/*w*) on the extraction of TPC was studied at 47 °C in the range from 10.9 to 35.3 mL:*g_BSG,dry_*. The results are presented in Figure 2a,b in terms of extract concentration, mg GAE/L, and extraction yield, mg GAE/g_BSG,dry_, respectively. Figure 2a shows that polyphenol concentration in the extract (mg GAE/L) increased as the ratio solvent: g_BSG,dry_ decreased in the range from 35.3 to 10.9 mL:g dry BSG. However, the extraction yield in terms of mg GAE/*g_BSG,dry_* reached a plateau from ratio values higher than 21.7 mL:*g_BSG,dry_*, while it decreased by using lower solvent:*g_BSG,dry_* ratios This can be also observed in Figure 3, where final extraction yield as mg GAE/*g_BSG,dry_* after 1800 s of extraction was plotted as a function of mL water:*g_dry BSG_* ratio together with the specific power density, W/*g_BSG,dry_*. By increasing the solvent:g_BSG,dry_ ratio, the specific power density also increased, observing a linear dependence, PD (J/s·g) = 1.055 (mL:*g_BSG,dry_*) + 0.191, R^2^ = 0.9983). Therefore, taking into account the TPC yield and the power density by UAE, the ratio 21.7 mL:*g_BSG,dry_* was fixed to study the effect of temperature and solvent type. 

In the literature, an increase in extraction yield was also observed by solvent bath extraction (maceration) for 60 min when using a 70% ethanol aqueous mixture at 60 °C with values of 2.59, 2.74, and 3.07 mg GAE/*g_BSG,dry_* at 10, 20, and 30 mL_solvent_/g_BSG,dry_, respectively [17]. These authors explained that higher liquid/solid ratio implies higher concentration gradient between the solid and the bulk of the liquid, resulting in a greater driving force for diffusion of compounds to the solvent and higher extraction efficiency. 

#### 3.2.3. Effect of Temperature on Polyphenol Extraction Kinetics by UAE

**Figure 4:**
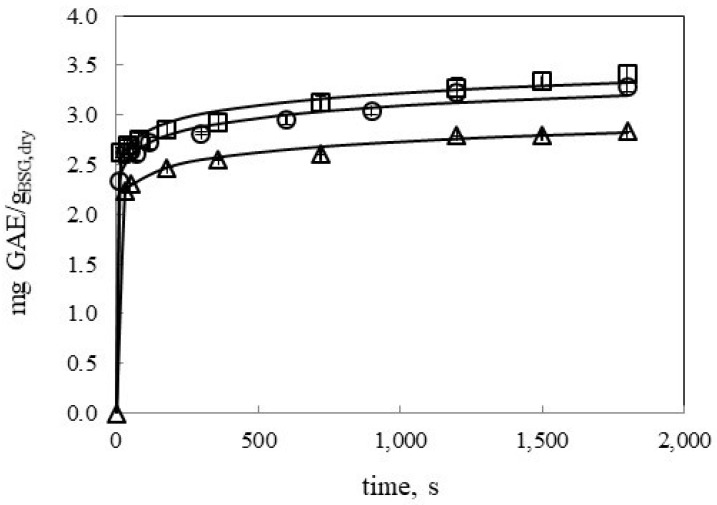
Effect of temperature on TPC water extraction kinetics by UAE (Δ 39 °C, ○ 47 °C; □ 58 °C) at a 21.7 mL:*g_BSG,dry_* ratio. Continuous lines represent the Weibull model.

In this work, TPC extractions by UAE have been carried out at three different temperatures, 39, 47, and 58 °C (Figure 4) at a ratio of 21.7 mL:*g_BSG,dry_*. Temperature affected positively TPC extraction kinetics by increasing temperature from 39 to 47 °C. Further increase in the temperature did not bring a faster kinetic extraction or higher extraction yield. An increase of temperature results in an increase of diffusivity and a decrease of viscosity and surface tension of the solvent. Therefore, faster extraction rate might be expected by increasing temperature. However, an increase in temperature also results in an increase of solvent vapor pressure that made more solvent vapor enter the bubble and collapse less violently reducing the sonication effects [7]. In this work, this effect was observed at the highest temperature essayed. Lower water viscosity and higher diffusivity with temperature were compensated by the increase of the solvent vapor pressure.

Other studies found in the literature reported temperature as one of the positive variables on the extraction of TPC from black chokeberry in the temperature range from 20 to 80 °C [16]. These authors also reported that the effect of temperature on kinetics of water extraction of TPC from black chokeberry was less significant for ground samples than for berries cut in half where extraction was not very efficient and solvent penetration and extraction were hampered [16]. 

#### 3.2.4. Effect of Ethanol Concentration on Polyphenol Extraction Kinetics by UAE

**Figure 5:**
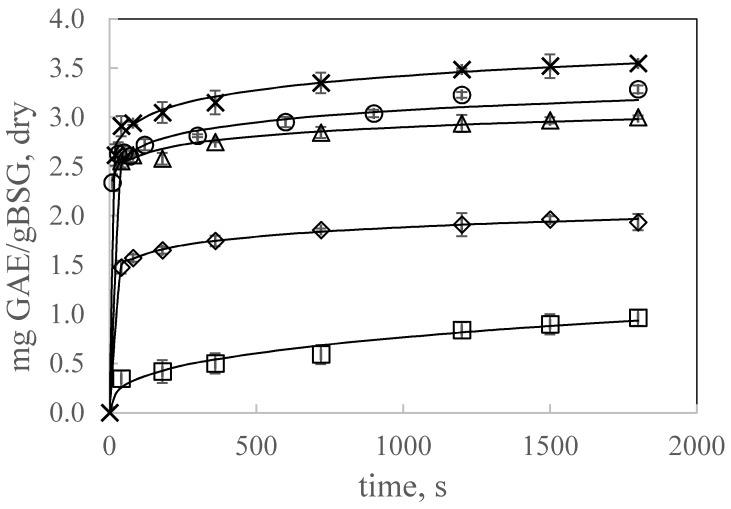
Effect of ethanol concentration on TPC extraction kinetics by UAE at 47 °C and 21.7 mL: g_BSG,dry_: ○ water, × 20% ethanol, △ 50% ethanol, ◊ 80% ethanol, □ 100% ethanol. Continuous lines represent the Weibull model.

In the literature, other solvents such as ethanol, methanol and acetone and their corresponding aqueous mixtures have been used to extract polyphenols from plant matrixes. In this work, different ethanol aqueous mixtures were also explored to determine the effect of solvent polarity on TPC extraction kinetics. Figure 5 shows the kinetics of TPC extraction from ground BSG using water, pure ethanol and different water-ethanol mixtures at 47 °C and 21.7 mL:*g_BSG,dry_*. TPC extraction was dependent on the type of solvent used and therefore on the polarity of the solvent. The best results were obtained for the hydroalcoholic mixture with 20% of ethanol. Pure water as solvent gave better extraction kinetics and higher extraction yields than pure ethanol or ethanol alcoholic mixtures with high ethanol percentage. Therefore, water could create a more polar medium that facilitates the extraction of phenolic compounds. Water and mixtures with low ethanol concentration could access to cells, but high concentration of ethanol could cause protein denaturation, preventing the dissolution of polyphenols, and then influencing the extraction [17]. Socaci et al. [2] found that an increase in ethanol concentration from 60% to about 68% increased TPC extraction while higher ethanol concentration led to a gradual decrease in TPC. The higher extraction efficiency for the 20:80 (EtOH:water) mixture could be attributed to the lower value of the dielectric constant for ethanol:water mixtures than the value for pure water that increases the solubility of phenolic compounds in hydroalcoholic mixtures with low ethanol content.

Dent et al. [18] carried out the study of TPC extraction from Dalmatian wild sage by conventional solvent extraction with ethanol and acetone aqueous mixtures and water as solvent. These authors found a maximum of total polyphenol content with 30% ethanol or acetone. However, these authors, concluded, that the differences in TPC between 30% ethanol and 50% acetone aqueous extracts with water extracts were not significant 

Therefore, in this work, taking into account the green chemistry principle, water was considered as an efficient solvent for TPC extraction from BSG.

#### 3.2.5. Kinetic Modelling

TPC extraction kinetics were fitted to different empirical models as described in Section 2.7. Table 3 lists the parameter of the models essayed. Both models, the power law and the Weibull models, fitted the data quite well with a mean RMSD for all the kinetics of 7.45 and 7.50, respectively.

For both models, the exponent of time, n, called the diffusion coefficient and the shape parameter for the power law and Weibull models, respectively, followed the same tendency. It presented low values for TPC extraction of ground BSG, with values lower than 0.1, except when using pure ethanol as solvent. The values of n of the power law model showed that Fickian diffusion controlled TPC extraction from BSG, while values of n < 1 for the Weibull model described the parabolic shape of the extraction curves with a high initial slope.

B and A parameters for the power law and the Weibull models, respectively, also followed the same tendency. These parameters presented a maximum at 20% w/w ethanol as solvent, increased with decreasing the particle size and decreased by decreasing the ratio solvent:BSG in the range covered in this work. Regarding k parameter for the Weibull model, it can be considered as the extraction rate constant having higher values for the fastest extraction kinetics. Figure 1, Figure 2, Figure 4 and Figure 5 include the Weibull model fitting, since it was considered a more realistic approach.

### 3.3. Determination of Extract Components

Table 4 shows the total amount of individual phenolic compounds released by UAE at 47 °C, 21.7 mL solvent:*g_BSG,dry_* by using water and 20% ethanol aqueous mixture as extraction solvents that could be identified by HPLC/DAD. By using water as solvent, concentration of identified individual phenolic compounds was not very high with values of 10.0 ± 0.5 µg of p-hydroxybenzoic acid/*g_BSG,dry_*, 10.7 ± 0.3 µg of ferulic acid/*g_BSG,dry_* and 2.8 ± 0.2 µg of sinapic acid/*g_BSG,dry_*. When using 20% ethanol as extraction solvent, similar results were obtained, although higher values for sinapic acid were quantified, 13.4 ± 0.3 µg of sinapic acid/*g_BSG,dry_*. 

**Figure 6:**
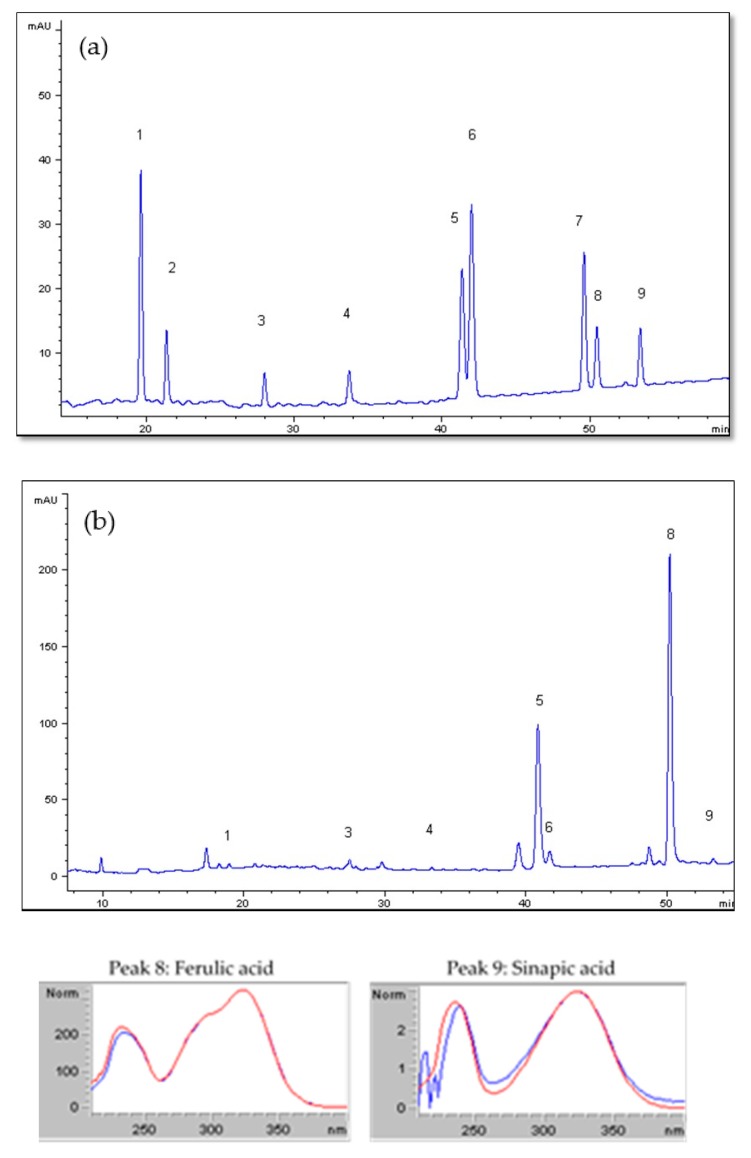
HPLC/DAD chromatograms at 280 nm of individual phenolic standards (**a**) and obtained after alkali hydrolysis of BSG (**b**). 1, p-Hydroxybenzoic acid; 2, protocatechuic aldehyde; 3, vanillic acid; 4, syringic acid; 5, p-coumaric acid; 6, vanillin; 7, syringic aldehyde; 8, ferulic acid; and 9, sinapic acid.

In this work, hydrolytic methods have been also carried out to compare the release of phenolic compounds (Table 4). The influence of the hydrolysis technique can be observed in the results of TPC and phenolic profile. As reported in Table 2, BSG used in this work contained 13.5% of insoluble lignin that it is connected to the cell wall polysaccharides by phenolic acids, being necessary a hydrolytic method to release them [19]. After acid hydrolysis, TPC was determined as 30 ± 5 mg GAE/*g_BSG,dry_*. This value is higher than the value obtained for basic hydrolysis, 16.2 ± 0.2 mg GAE/*g_BSG,dry_*. In the literature, it has been reported that acid hydrolysates of wheat flour and wheat bran yielded higher total phenolic content than the corresponding alkali hydrolysates [10], similar to the results obtained in this work. Furthermore, the amount of individual phenolic compounds released in each extraction technique was different. For basic hydrolysis 1305.7 ± 0.5 µg ferulic acid/g dry BSG were released while only 54.4 ± 0.3 by acid hydrolysis. The same trend was observed in the acid and basic hydrolysis of wheat bran by Arranz and Saura Calixto [10], 219 ± 5 and 60 ± 2 mg ferulic acid/100 g of fresh weight wheat bran by basic and acid hydrolysis, respectively. The p-coumaric acid/ferulic acid ratio (2.43) obtained for basic hydrolysis was consistent with the values reported in the literature (1.74–2.97) [20]. Phenolic acids esterified to the cell wall were more easily released under basic conditions, due the higher solubility of the lignin under basic conditions. HPLC chromatograms for phenolic standards and basic hydrolysis are presented in Figure 6. The percentage match obtained comparing the UV spectra of authentic standards with the UV spectra of the selected peaks were the following: p-hydroxybenzoic acid 95.8%, vanillic acid 98.83%, syringic acid 90.29%, p-coumaric acid 99.99%, vanillin 99.16%, ferulic acid 99.6%, and sinapic acid 90.21%. Protocatechuic aldehyde and syringic aldehyde were not identified in any of the extracts.

Moreira et al. [21] determined the TPC of different types of BSG with a maximum content of 20 mg GAE/*g_BSG,dry_* as determined by Folin–Ciocalteau after microwave assisted extraction for 0.75% NaOH concentration. Total alkali-extractable ferulic acid and p-coumaric acid was reported as 1.8 mg ferlulic acid/*g_BSG,dry_* and 0.8 mg p-coumaric/g_BSG,dry_ from the BSG obtained from Mahou S.A [20]. Most of the literature studies have reported p-coumaric and ferulic acids as the most abundant hydroxycinnamic acids in BSG [6]. However, other authors found that the two main phenolic compounds were p-hydroxybenzoic and protocatechuic acid, by using BSG as by product from the mashing process of dark larger beer [2]. As a general trend, lower amounts of hydroxycinamic acids have been reported in different aqueous methanolic extract when comparing pale and black BSG [6]. In this work, p-hydroxybenzoic was determined, but protocatechuic acid was not detected.

Table 4 also lists the TPC and the identified individual phenolic compounds obtained by enzymatic hydrolysis with a commercial xylanase. It can be observed that an increase in the xylanase concentration (*w*/*w*, referred to BSG), led to an increase of TPC extraction yield. However, at 6% *w*/*w* of xylanase, a release of 23% and 1% of total alkali-extractable ferulic acid p-coumaric acid, respectively, was only obtained. Bartolomé and Gómez-Cordovés [20] determined the release of ferulic and p-coumaric acid by different commercial enzyme preparations with ferulic acid esterase activity. These authors also found that for the most active enzyme preparation (Lallzyme preparation) the enzymatic release of these two hydroxycinnamic acids was lower than for the alkali extraction, especially for p-coumaric acid, reaching maximum values around 70% and 10% with respect to the basic hydrolysis for ferulic and p-coumaric, respectively. It can be concluded that basic conditions are needed to release the hydroxycinnamic acids. 

Productivity of the different extraction or hydrolytic methods after 30 min of treatment has been listed in Table 4. Compared to xylanase hydrolysis, higher or similar values were obtained by UAE after 30 min of treatment. The highest productivity value at 30 min was obtained by alkali hydrolysis. However, the use of NaOH results in a more aggressive extraction medium.

For UAE water extracts individual sugars and soluble protein concentrations were also determined. Individual sugars concentrations were 9.4 mg glucose/*g_BSG,dry_*, 31.7 mg xylose/*g_BSG,dry_* and 21.8 mg arabinose/*g_BSG,dry_*. Therefore, total arabinoxylans (AX) content in the extract was 53.5 mg AX/*g_BSG,dry_*_,_ reaching 18% extraction yield of AX at the selected extraction conditions determined for TPC extraction. The efficiency of UAE in the production of AX-rich extracts from BSG was studied by Reis et al. [22] finding that a maximum of 4.8% yield could be achieved for water soluble AX for 12 min of ultrasound treatment, 92% maximum amplitude and 23 min of autoclave treatment at 120 °C after ultrasound treatment for a 25 mL solvent/g_BSG,dry_ ratio. The higher yield obtained in the present work could be attributed to longer ultrasound treatment time, 30 min. In any case, these authors concluded that the highest yield, 60%, was achieved, when alkaline ultrasound treatment was applied.

The concentration of soluble protein in UAE water extracts was 82 ± 1 mg protein/*g_BSG,dry_*. This value is of the same order as the one reported by Tang et al. [23] in the study of UAE of protein fraction from BSG, with an optimum yield value of 104.2 mg protein/*g_BSG._*

Total yield of UAE water extract was determined by weighting the extract after removing all the solvent by evaporation in an oven at 105 °C until constant weight, a value of 24 g of dry extract/100 *g_BSG,dry_* was obtained. With this value, concentrations could be easily expressed in a dry extract basis instead of per gram of dry BSG, when needed.

### 3.4. Partial Concentration by Centrifuge Ultrafiltration

**Figure 7:**
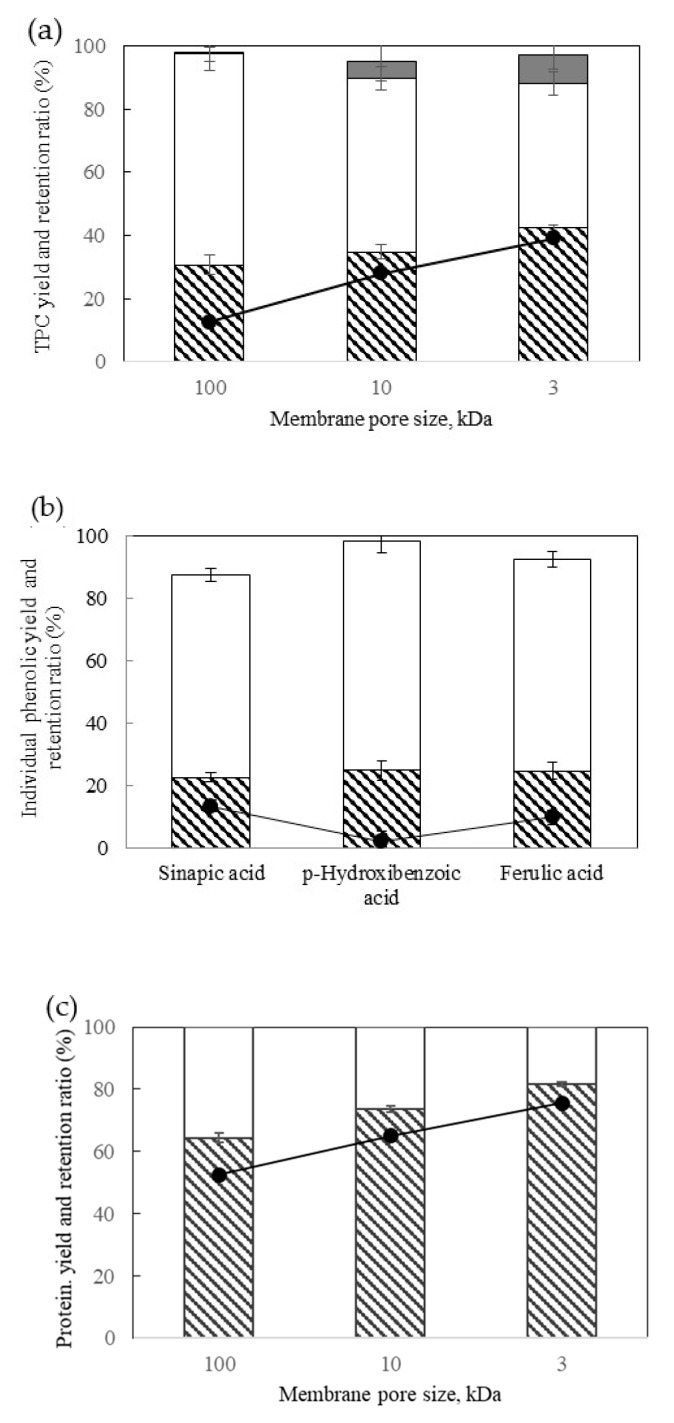
Permeate/retentate yield and retention ratio at different membrane NMWL (**a**) TPC (**b**) individual phenolic compounds at 10 kDa membrane size (**c**) protein. Columns represent the yield (▧ rententate yield, □ permeate yield, ■ TPC adsorbed in the membrane), ● retention ratio.

The extract obtained by water solvent extraction by UAE at 47 °C for a 21.7 mL:*g_BSG,dry_* ratio was submitted to centrifuge ultrafiltration to partially concentrate the fraction of the total polyphenol compounds. Figure 7a shows that the TPC yield in the retentate slightly increased by decreasing the NMWL of the membrane from 31% to 41% for 100 and 3 kDa, respectively, and so it did the retention ratio. The regenerated cellulose membrane allowed a high recovery of TPC, not being adsorbed in the membrane, however, it can be observed an increase in the TPC adsorbed in the membrane (as recovered by MeOH after centrifuge ultrafiltration) by decreasing the NMWL of the membrane, reaching values up to 9% for 3 kDa. For the 10 kDa membrane, Figure 7b shows the permeate and retentate yield of the different phenolic compounds identified by HPLC DAD. Ferulic acid, sinapic acid and p-hydroxybenzoic acid preferably crossed the membrane and concentrated on the permeate, as can be also observed in the low values of the retention ratio.

The separation and concentration results of the co-extracted soluble protein fraction have been plotted in Figure 7c. The protein yield in the retentate increased by decreasing the NMWL of the membrane from 64% to 82% and a retention coefficient for protein from 53% to 76% for 100 and 3 kDa respectively. Tang et al. [13] in the study of protein concentration from BSG reported that more than 92% protein was retained by ultrafiltration membranes with molecular weight cut off of 5 and 30 kDa. 

The separation and concentration of the co-extracted individual sugars have been also determined, reaching sugars yields up to 65% in the permeate for the different NMWL of the membrane studied. Therefore, it can be concluded that by centrifuge ultrafiltration with membranes of NMWL lower than 10 kDa, a permeate rich in hydroxycinnamic acids and sugars can be obtained while the retentate will be enriched in the protein fraction. 

## 4. Conclusions

A complete brewery spent grains valorization should first include extractives valorization due to the important amount of these easily extractable compounds and the presence of important bioactive compounds, such as hydroxycinnamic acids. The analysis of the data led to the following conclusions. UAE has been shown to be more efficient in the total polyphenol extraction than conventional extraction. The most significant operating parameters were particle size and type of solvent. Water has been proved as a good solvent for total polyphenol extraction meeting the green principle extraction. The power law and the Weibull models fitted experimental data quite well. Compared to other hydrolytic extraction methods, UAE productivity after 30 min of treatment yielded similar values as enzyme hydrolysis. However, UAE has been shown to be not as effective as basic hydrolysis to release the phenolic compounds esterified to the cell wall. By a further concentration step via centrifuge ultrafiltration a retentate rich in the soluble protein fraction can be obtained while low retention ratios were obtained for the individual phenolic compounds and sugars identified in the extracts.

The results obtained in this work are useful as a preliminary step in the industrial design of a complete valorization of the BSG since large-scale ultrasonic equipment are available.

## Figures and Tables

**Table 1:** Particle size distribution of the original brewer’s spent grain (BSG) and ground BSG.

Original BSG		Ground BSG	
Size, mm	Mass Percentage, %	Size, mm	Mass Percentage, %
>4	4.4	>1	2.9
2–4	84.5	0.5–1	31.3
1–2	10.3	0.25–0.50	34.8
0.5–1	0.71	0.125–0.25	24.6
0.25–0.50	0.06	<0.125	6.5

**Table 2:** Chemical composition of BSG from a local Spanish brewery.

Component	g/100 *g_BSG,dry_*
Extractives in water	24.3 ± 0.6
Extractives in ethanol	1.8 ± 0.2
Starch	7.9 ± 0.2
Cellulose	18.2 ± 1.6
Hemicellulose	26.1 ± 1.8
Insoluble Lignin	13.5 ± 0.5
Soluble lignin	4.3 ± 0.1
Proteins	17.8 ± 0.1
Lipids	5.90 ± 0.01
Ash	2.92 ± 0.02

**Table 3:** Kinetic model parameters for TPC extraction from BSG.

Extraction Mode	Pretreatment	T, °C	Solvent	mL: *g_BSG,dry_*	Power Law Model			Weibull Model			
					B	n	RMS	A	k	n	RMS
MS	-	47	Water	21.7:1	0.208	0.264	5.74	5.2450	0.027	0.341	4.88
UAE	-	47	Water	21.7:1	0.431	0.241	7.32	4.1643	0.088	0.319	6.56
MS	ground	47	Water	21.7:1	1.197	0.097	5.23	6.133	0.197	0.127	6.37
UAE	ground	47	Water	21.7:1	2.066	0.057	6.47	5.895	0.415	0.084	6.80
UAE	ground	47	Water	35.3:1	2.027	0.057	8.89	5.699	0.429	0.080	10.10
UAE	ground	47	Water	18.1:1	1.744	0.065	5.97	5.120	0.4070	0.090	6.52
UAE	ground	47	Water	13.6:1	1.297	0.097	8.94	5.796	0.244	0.125	9.40
UAE	ground	47	Water	10.9:1	1.438	0.084	9.22	4.643	0.391	0.096	5.92
UAE	ground	39	Water	21.7:1	1.837	0.057	20.68	6.664	0.316	0.075	21.82
UAE	ground	58	Water	21.7:1	2.159	0.058	9.66	6.418	0.403	0.079	9.93
UAE	ground	47	20% EtOH	21.7:1	2.229	0.062	4.92	8.144	0.314	0.080	4.97
UAE	ground	47	50% EtOH	21.7:1	2.110	0.046	4.37	4.830	0.559	0.072	4.59
UAE	ground	47	80% EtOH	21.7:1	1.130	0.074	1.59	3.458	0.376	0.107	1.52
UAE	ground	47	100% EtOH	21.7:1	0.073	0.342	5.35	3.491	0.019	0.372	5.68

MS: mechanical stirring, UAE: ultrasound assisted extraction.

**Table 4:** Individual phenolic compounds in the different extracts (µg/g dry BSG).

Compounds	Formula	UAE-W	UAE-20EtOH	Acid-Hydrolysis	Alkaline-Hydrolysis	Xylanase 1%	Xylanase 3%	Xylanase 6%
p-hydroxybenzoic acid	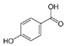	10.0 ± 0.5 ^a^	10.0 ± 0.7 ^a^	n.d.	59.3 ± 2.2 ^b^	n.d.	n.d.	n.d.
Vanillic acid	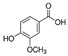	n.d.	n.d.	n.d.	48.8 ± 1.5 ^c^	17.9 ± 1.1 ^a^	42.6 ± 2.3 ^b^	61.2 ± 3.3 ^d^
Syringic acid	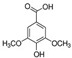	n.d.	n.d.	n.d.	106.1 ± 5.7	n.d.	n.d.	n.d.
p-Coumaric acid	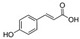	n.d.	n.d.	n.d.	538.2 ± 4.4 ^b^	5.9 ± 1.4 ^a^	5.3 ± 1.7 ^a^	5.3 ± 0.4 ^a^
Vanillin	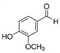	n.d.	n.d.	n.d.	217.2 ± 1.4 ^c^	110.5 ± 2.6 ^a^	191.2 ± 0.1 ^b^	203.5 ± 10 ^b,c^
Ferulic acid	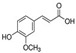	10.7 ± 0.3 ^a^	9.5 ± 0.3 ^a^	54.4 ± 0.3 ^b^	1305.7 ± 0.5 ^e^	52.4 ± 0.9 ^b^	185.8 ± 4.5 ^c^	292.4 ± 2.6 ^d^
Sinapic acid	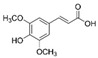	2.8 ± 0.2 ^a^	13.5 ± 0.3 ^c,d^	31.1 ± 0.5 ^f^	27.2 ± 1.2 ^e^	7.5 ± 0.2 ^b^	12.9 ± 0.6 ^c^	14.9 ± 1.2 ^d^
TPC (Folin-Cioculteau) mg GAE/g_BSG,dry_·min		3.28 ± 0.12 ^a^_0.5h_	3.55 ± 0.07 ^a^_0.5h_	30 ± 5 ^e^_24h_	16.2 ± 0.2 ^c^_4h_	7.2 ± 0.2 ^b^_24h_	25.2 ± 0.1 ^d^_24h_	42.0 ± 0.4 ^f^_24h_
Productivity, mg GAE/g_BSG,dry_·min*		0.109 ± 0.002 ^a,b^	0.118 ± 0.004 ^b^	non-determined	0.55 ± 0.04^c^	0.050 ± 0.004 ^a^	0.065 ± 0.002 ^a,b^	0.087 ± 0.001 ^a,b^

n.d.: non detected. Values with different letters (a–f) in each raw are significantly different when applying the Fisher’s least significant differences (LSD) method at *p*-value ≤ 0.05. * evaluated after 30 min of extraction.
